# Piezo1 Ion Channels Regulate the Formation and Spreading of Human Endometrial Mesenchymal Stem Cell Spheroids

**DOI:** 10.3390/ijms26062474

**Published:** 2025-03-10

**Authors:** Zuleikha M. Khairullina, Valeria Y. Vasileva, Vladislav I. Chubinskiy-Nadezhdin

**Affiliations:** Institute of Cytology, Russian Academy of Sciences, Tikhoretsky Ave. 4, 194064 St. Petersburg, Russia

**Keywords:** Piezo1, mechanosensitive channels, mesenchymal stem cells, cell spheroids, Yoda1, calcium signaling, cell migration

## Abstract

Mesenchymal stem cells obtained from desquamated endometrium (eMSCs) are considered as reliable and promising objects for stem cell-based therapy. eMSCs aggregated into three-dimensional (3D) spheroids demonstrate greater efficiency compared to monolayer 2D eMSCs. However, molecular processes and specific mechanisms regulating the effectiveness of spheroids remain unknown. Regulation of a number of physiological reactions in MSCs is associated with the functioning of Ca^2+^-permeable mechanosensitive Piezo1 channels. In our previous study, we showed that selective Piezo1 activation by its selective agonist Yoda1 controls the migratory activity of 2D eMSCs. Here, we aimed to determine the effect of Yoda1 on eMSC spheroid formation and spreading. *PIEZO1* mRNA expression was lower in spheroids compared to 2D culture. Spheroids formed with Yoda1 or spread in the presence of Yoda1 demonstrated lower spreading rates compared to control (Yoda1-free) spheroids. The spreading rates of control spheroids depended on the substrate stiffness, whereas spheroids formed with Yoda1 had similar spreading rates regardless of the surface properties. Our results demonstrate several Piezo1-dependent reactions of eMSC spheroids that could be modulated by selective Piezo1 activation.

## 1. Introduction

Three-dimensional (3D) cultivation of mesenchymal stem cells (MSCs) as spheroids allows more accurate imitation of complex cell–cell and cell–extracellular matrix interactions that occur in vivo [1,2]. Spheroids formed from MSCs also demonstrate significantly higher efficiency in various stem cell therapies compared to cells cultured as monolayers (i.e., traditional 2D cultivation on cell culture plastic) [3,4]. However, there are many open questions on the nature and characteristics of molecular biological processes occurring in 3D structures. Particularly, the specific pathways that could affect spheroid formation, or interactions between the spheroids and its environment in the course of spheroid-based therapy, remain unresolved.

It is known that the regulation of important biological processes, such as proliferation, migration, adhesion, etc., in particular, is associated with the activity of mechanosensitive ion channels, especially those that can mediate the influx of calcium ions, ubiquitous second messengers, into the cell [5]. According to several studies, the mechanosensitive Piezo1 channel is considered one of the main mechanosensors in different types of cells, including MSCs of various origins, such as bone marrow-derived MSCs, adipose-derived MSCs, dental-derived MSCs, human umbilical cord MSCs, etc. [6,7,8,9,10,11,12,13,14]. It is obvious that the processes of cell aggregation into 3D structures and disaggregation of 3D structures (spheroid spreading) are also based on mechanotransduction processes, in which Ca^2+^-permeable mechanosensitive Piezo1 channels may be possibly involved. However, questions remain open regarding their participation and role in the spatial and temporal regulation of calcium signaling and in other cellular processes in 3D aggregates. It is important to note that the role, expression level, and functional properties of ion channels can vary significantly with different types of cell culturing (2D or 3D). Evidently, cell aggregation into spheroids is accompanied with drastic alterations in cell shape, size, plasma membrane curvature, and cytoskeleton structure [15], which in turn could be regulated by or regulate Piezo1-dependent signaling pathways.

Previously, using the selective chemical activator Yoda1 [16], we identified Piezo1 channels in endometrial MSCs (eMSCs) as one of the key regulators of intracellular Ca^2+^ controlling cell migration [17]. The main advantages of eMSCs are non-invasive isolation protocols and a lack of significant ethical issues that favor their use in stem cell-based therapies [18]. Importantly, eMSC spheroids were shown to increase their therapeutic potential compared to 2D eMSCs [3]. Taken together, in the current study we aimed to determine the functional expression and the role of Piezo1 in eMSC spheroids and to compare the effect of selective Piezo1 activation between 2D and 3D eMSC cultures. Particularly, we probed whether functional Piezo1 was expressed in the plasma membrane of the spheroids and quantified the differences in *PIEZO1* mRNA expression between 2D cells and spheroids. Further, we identified whether selective chemical Piezo1 stimulation by its agonist, Yoda1, affected the formation, viability, and spheroid spreading rates. Finally, we compared the spheroid spreading after selective Piezo1 activation on culture plastic and glass, surfaces of different stiffness.

## 2. Results and Discussion

### 2.1. Immunofluorescent Staining and Polymerase Chain Reaction (PCR) Confirm the Presence of Piezo1 in eMSC Spheroids

**Figure 1:**
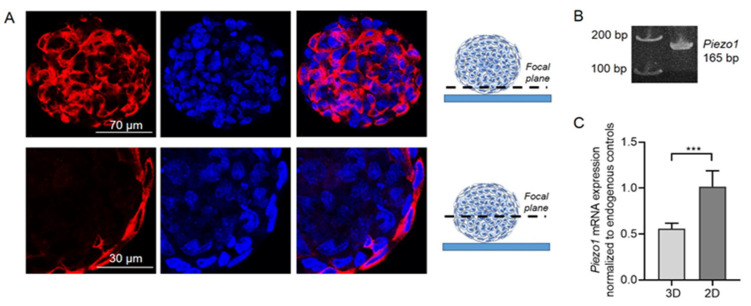
Expression of Piezo1 in eMSC spheroids. (**A**) Immunofluorescent staining evidenced the presence of Piezo1 proteins. Shown are representative confocal slices obtained from the bottom plane (near the cover glass) and through the center of the spheroid. The schematic illustrations of focal planes used for acquisition are shown at the right from the images. Red channel—Piezo1 immunofluorescence, blue channel—cell nuclei. Note that in the focal plane through the center no Piezo1 staining is observed in the cells inside the spheroids, which is presumably due to the inability of the antibodies to penetrate inside the spheroid mass as well as the used antibodies not requiring permeabilization. No staining was observed if the primary antibodies were omitted (Supplementary Figure S1). (**B**) *PIEZO1* mRNA was detected in spheroid lysates: the primers against *PIEZO1* amplified the product of the expected size (165 bp). Original gels are shown in Supplementary Figure S2 (**C**) Relative mRNA expression levels of *PIEZO1* in 2D and 3D eMSC cultures counted using the 2^−ΔΔCt^ method. The qPCR data were normalized to *HPRT1* and *TBP* gene mRNA (see Section 3). ***—significantly different, unpaired *t*-test, *p* < 0.001.

Firstly, we performed immunofluorescent staining using specific anti-Piezo1 antibodies that clearly evidenced the presence of Piezo1 proteins in the eMSC spheroids (Figure 1A). Consistently, conventional PCR confirmed the presence of *PIEZO1* mRNA in spheroid lysates (Figure 1B). Next, using qPCR, we compared the levels of *PIEZO1* mRNA expression between eMSC spheroids and 2D eMSC cultured as monolayer cell culture. Importantly, we observed a decrease in *PIEZO1* mRNA content in spheroids compared to 2D cell culture by approximately 1.8-fold (Figure 1C). These results are in good correlation with our previous report, where, using single-channel patch-clamp analysis, we evidenced a significant decrease in the frequency of activation of stretch-activated channels (with biophysical characteristics similar to Piezo1) in eMSC spheroids compared to 2D cell culture [19]. It is of specific interest why the cells in eMSC spheroids decreased the amount of *PIEZO1*, and it could be the goal for further studies. We could speculate that the changes in gene expression might be due to the changes in physical microenvironment and cell morphology.

### 2.2. Selective Chemical Piezo1 Activation Induces Ca^2+^ Entry in eMSC Spheroids

**Figure 2:**
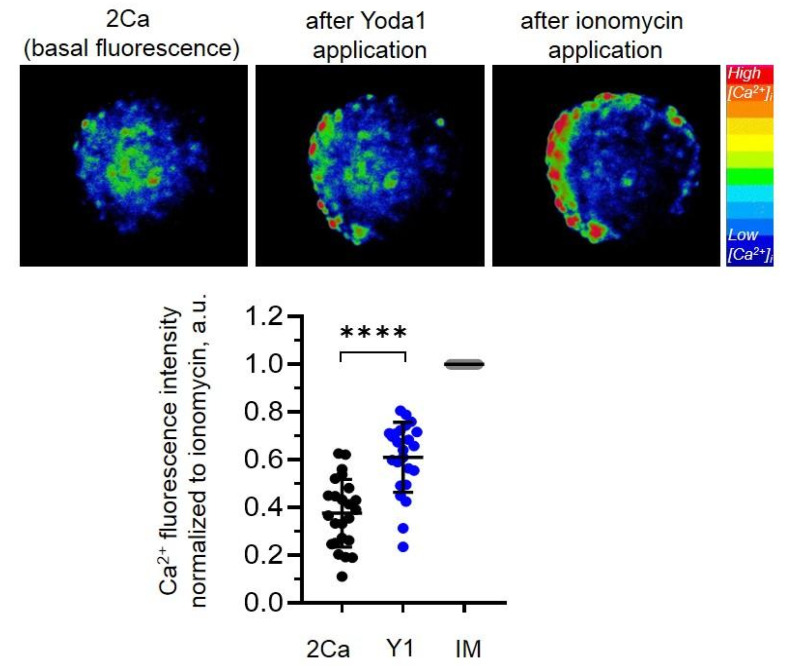
Selective chemical Piezo1 agonist Yoda1 evokes Ca^2+^ entry in eMSC spheroids. Shown are pseudo-colored images of the representative spheroid loaded with Fluo8 Ca^2+^ probe demonstrating the increase of [Ca^2+^]_i_ after application of 10 µM Yoda1. Ca^2+^ ionophore ionomycin (IM) was applied at the end of each experiment to evoke maximal Ca^2+^ entry (maximal Fluo8 fluorescence). The level of basal fluorescence (in 2Ca, before Yoda1 addition) and fluorescence after Yoda1 application (Y1) of each cell was normalized to the maximal Ca^2+^ entry (fluorescence after ionomycin, IM). Shown are normalized mean (±S.D.) fluorescence values of n = 24 cells from n = 3 independent experiments (individual spheroids). ****—significantly different, paired *t*-test, *p* < 0.0001.

The presence of functionally active Piezo1 channels in the plasma membrane of the cells on the surface of the eMSC spheroids was probed in Ca^2+^ imaging experiments. In particular, we applied a selective chemical Piezo1 activator, a small synthetic compound Yoda1 [16], and monitored the changes in internal Ca^2+^ concentration ([Ca^2+^]_i_) in eMSC spheroids. Previously, we determined the specific Yoda1 concentration that induced Ca^2+^ influx and influenced cell migration and proliferation of 2D eMSCs but had no effect on cell viability [17]. Here, we used the same concentration of Piezo1 agonist to stimulate the channels in eMSC spheroids: we detected that 10 µM Yoda1 induced the increase in [Ca^2+^]_i_, thus clearly indicating the presence of functional Piezo1 in the plasma membrane (Figure 2). Thus, eMSC spheroids (despite the observed decrease in *PIEZO1* mRNA expression) retain functionally active Piezo1 channels, whose activity could be evoked by Yoda1.

### 2.3. Role of Piezo1 Activity in Formation of eMSC Spheroids

**Figure 3:**
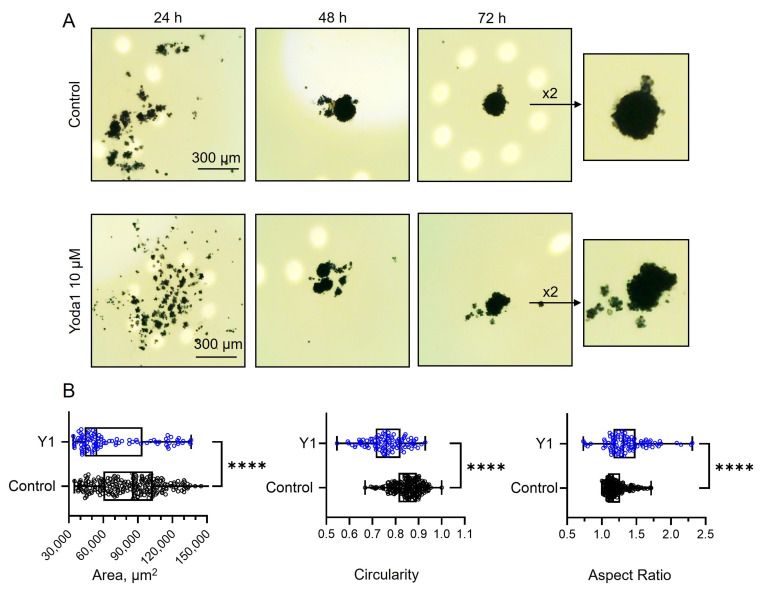
The presence of Yoda1 in the hanging drops during eMSCs spheroid formation affects the morphological parameters of the spheroids. (**A**) Shown are the cells in different stages of spheroid formation at 24, 48, and 72 h in control (0.1% DMSO) and in the presence of 10 µM Yoda1. Note the dark color of the spheroids that is due to the MTT reaction, thus indicating that the cells in hanging drops were viable (see Materials and Methods for details). (**B**) The morphological parameters of the spheroids after 72 h. The exposure of eMSCs to Yoda1 resulted in the formation of smaller (lower area) and more irregular (less circular and more elongated) spheroids, compared to control. Shown are medians (inside the box), upper and lower quartiles (box borders), and minimal and maximal values (whiskers). The area, circularity, and aspect ratio parameters were significantly different between the groups (**** *p* < 0.0001, Mann–Whitney test). Total of n = 230 control and n = 98 spheroids formed with Yoda1 were analyzed.

Next, we aimed to investigate how the selective Piezo1 activator Yoda1 affected the process of spheroid formation. Figure 3A shows the representative images of the cells in hanging drops at different stages of cell aggregation in spheroids. At the final time point, control cells formed round-shaped spheroids, whereas Yoda1-treated cells formed spheroids of irregular shape and significantly smaller sizes (Figure 3B). We speculate that the mechanism of the effect of Yoda1 on spheroid shape could be linked with the perturbation of the interactions of the cells during spheroid compactization. Interestingly, two molecules that were shown to participate in the processes of spheroid formation, integrins and cadherins [20,21], were shown to be regulated by Piezo1 activity [22,23,24]. It should be noted that the unequivocal evidence of the proposed mechanism of spheroid formation regulation by Piezo1 is difficult to reliably obtain.

Inclusion of Yoda1 to hanging drops during spheroid formation could induce prolonged Ca^2+^ influx via Piezo1 altering Ca^2+^ homeostasis that results in cell death. Thus, it is of specific importance to probe the viable status of the cells in hanging drops during spheroid formation to exclude the possibility that the effects of Yoda1 were due to the loss of cellular viability. The inclusion of MTT reagent to the drops clearly demonstrated that the spheroids in Yoda1-containing hanging drops are indeed viable: the addition of an MTT reagent resulted in dark color (see Section 3) due to the processing of MTT to the violet product formazan by the mitochondria of living cells (note the color of the spheroids shown in Figure 3A). Thus, selective Piezo1 activation during spheroid formation did not prevent the process nor affected cell viability, but resulted in the change of spheroid morphology. In contrast, in our recent study we have shown that Yoda1 completely blocked the formation of spheroids from an aggressive melanoma cell line, which was due to cell death in response to selective Piezo1 activation [25].

### 2.4. Piezo1 Activation Decrease the Rate of eMSCs Spheroid Spreading

**Figure 4:**
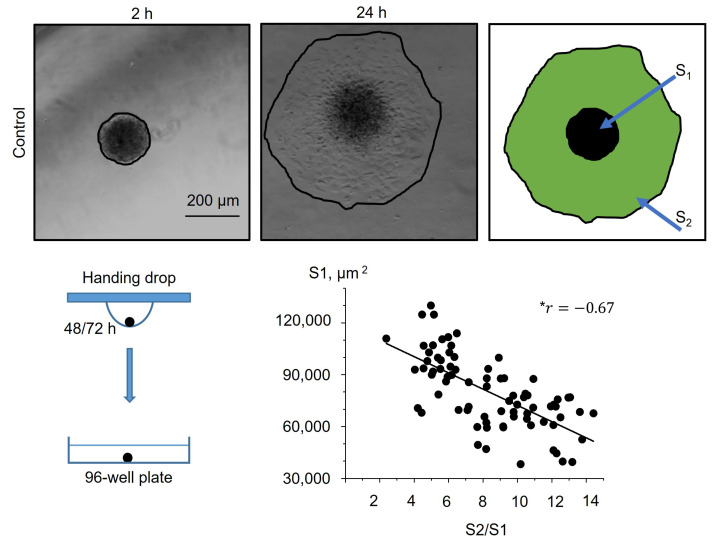
Dependence of the eMSC spheroid spreading rate (S2/S1) on the initial size of the spheroid (S1). Shown are representative images after 2 h from spheroid seeding to the well and after 24 h from the start of the experiment. Spheroid borders are designated by the black lines, and S2 and S1 areas are shown as a merged image. Note the strong negative correlation between S1 and S2/S1. * *r* is the Pearson linear correlation coefficient. n = 78 spheroids were quantified.

Further, we studied how selective Piezo1 activation affected the process of cell spreading from eMSC spheroids. We used two experimental protocols: (1) spheroids that were formed under control conditions (0.1% DMSO) were seeded to the wells of a 96-well plate and then 10 µM Yoda1 was added to the culture media (see the scheme of the protocols in Section 3) and (2) spheroids that were formed with Yoda1 in the hanging drops were plated to the wells in full culture media without Yoda1. As a control, eMSC spheroids formed with 0.1% DMSO were plated in full culture medium without DMSO washout (Figure 4). As the spheroids varied in initial size (S1), firstly we analyzed how spheroid spreading rate (S2/S1) was dependent on S1 value, and we observed a strong negative correlation between S1 and S2/S1 (Figure 4): i.e., the small spheroids spread faster than large ones. Thus, these results should be taken into account for the analysis of spheroid spreading rates: the spheroids of close initial sizes (i.e., S1 areas) should be selected from the datasets for proper comparison between control and Yoda1-exposed eMSC spheroids.

**Figure 5:**
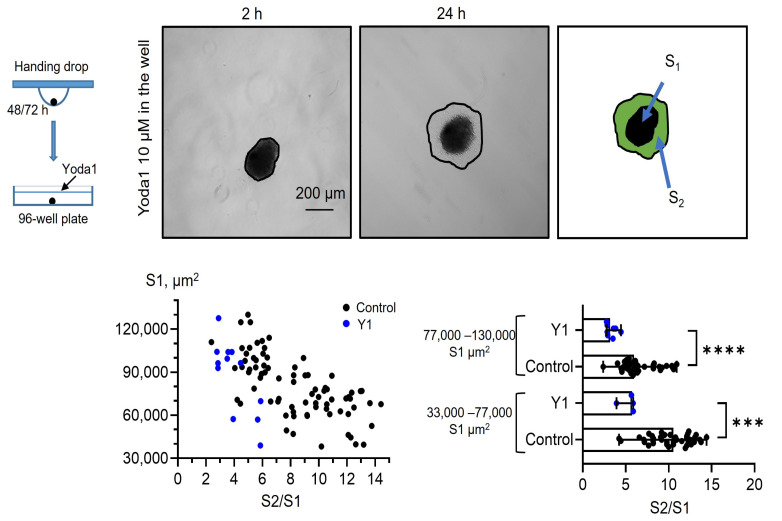
Yoda1-treated eMSC spheroids have significantly lower spreading rates. Shown are representative images after 2 h from spheroid seeding to the well and after 24 h from the start of the experiment. Spheroid borders are designated by the black lines, and S2 and S1 areas are shown as a merged image. The S2/S1 vs. S1 data points are plotted for control and Yoda1-treated spheroids as black and blue dots, respectively. Bar graphs showing medians with range and each point in the dataset. ****—*p* < 0.0001, ***—*p* < 0.001, significantly different, Mann–Whitney test. Total n = 78 control and n = 12 Yoda1-treated spheroids were analyzed.

**Figure 6:**
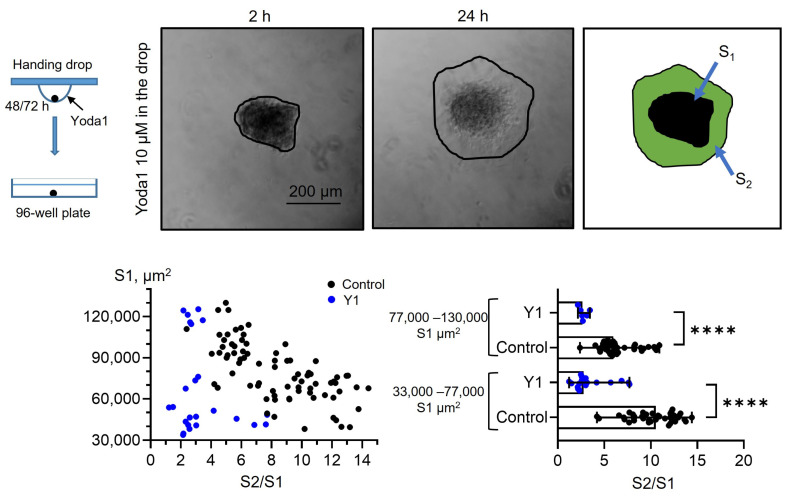
eMSC spheroids formed in the presence of Piezo1 activator Yoda1 have lower spreading rates. Shown are representative images after 2 h from spheroid seeding to the well and after 24 h from the start of the experiment. Spheroid borders are designated by the black lines, and S2 and S1 areas are shown as a merged image. The S2/S1 vs. S1 data points are plotted for control spheroids and spheroids formed in the presence of Yoda1 as black and blue dots, respectively. Bar graphs showing medians with ranges and all experimental points in the dataset. **** significantly different, *p* < 0.0001, Mann–Whitney test. Total of n = 78 and n = 24 control and Yoda1-treated spheroids were analyzed, respectively.

Taking this into account, we divided control, Yoda1-treated spheroids, and spheroids formed in the presence of Yoda1 into the two experimental groups based on their median initial size S1 (i.e., median S1 value in control), and compared the S2/S1 values between control and Yoda1-exposed groups. We observed a significant decrease in the spreading rate of the spheroids in the presence of Yoda1 during spreading or formation (Figure 5 and Figure 6), and this decrease was abolished in the presence of GsMTx4 [26], a general inhibitor of mechanosensitive cationic channels, including Piezo1 (Supplementary Figure S3). Also, even small spheroids that were formed in the presence of Yoda1 had lower spreading rates than larger spheroids formed under control conditions: i.e., the smaller size of the spheroid could not compensate for the drastic effect of Yoda1 on cell spreading. Thus, irrespective of the stage at which the spheroids were treated with selective Piezo1 activator, these spheroids possess lower spreading rates compared to control. The decrease of wound healing speed of canonical two-dimensional eMSC culture in the presence of Yoda1 was previously reported in our study [17]. Interestingly, dental-derived MSCs were shown to increase their migration rates after selective Piezo1 activation [10]. Most likely, the role of Piezo1 in cell migration could be determined by the composition of Piezo1-regulated signaling pathways or other factors that control Piezo1 properties [27].

### 2.5. Piezo1 Activation Eliminates the Differences in eMSC Spreading Rates on Plastic and Glass Surfaces

**Figure 7:**
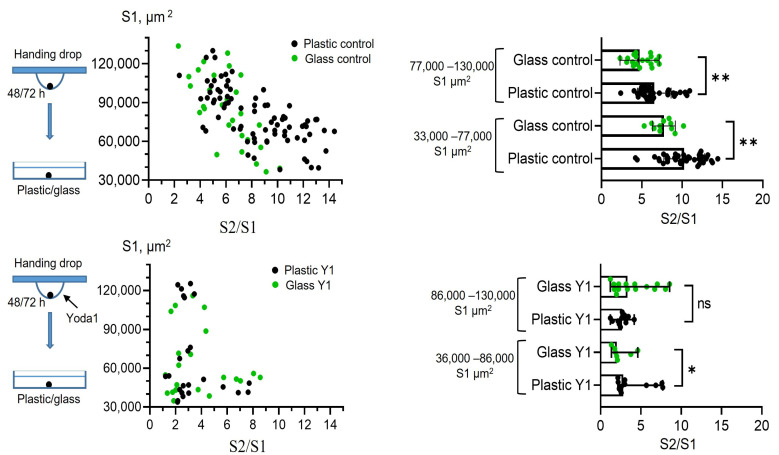
eMSC spheroid spreading rates on plastic and glass surfaces is dependent on Piezo1 activity. On the left, the S2/S1 vs. S1 data points are plotted for spheroids formed under control conditions (control) and in the presence of Yoda1 (Y1) spread on plastic (plastic control and Y1, black dots) and glass (glass control and Y1, green dots). Note almost similar distributions of experimental values for the spheroids formed with Yoda1 and spread on glass and plastic (plastic Y1 and glass Y1). Yoda1-formed spheroids were divided into two experimental groups, based on the median size of the spheroid area (S1). On the right, corresponding quantifications of S2/S1 rates. Bar graphs showing medians with ranges and all experimental points in the dataset. ns—non-significantly different, * *p* < 0.05, ** *p* < 0.01. Mann–Whitney test. Total number of control spheroids spread on plastic (n = 78) or glass (n = 30); Yoda1-treated spheroids spread on plastic (n = 24) or glass (n = 22).

Finally, as Piezo1 was shown to perform as a sensor of substrate stiffness [28,29], we aimed to compare how the rates of spreading of control spheroids and the spheroids formed in the presence of Yoda1 were dependent on the stiffness of the surface. Particularly, we seeded the spheroids on the culture plastic and on the glass slides, and calculated S2/S1 rates for all experimental conditions (Figure 7). The control spheroids (i.e., formed without Yoda1) had significantly lower spreading rates on stiffer substrate (glass) compared to softer culture plastic. This demonstrates a negative correlation between the rate of cell spreading (S2/S1) and the stiffness of the substrate. Thus, the spheroids from eMSCs can be classified as “substrate stiffness-dependent” cells [30]. It is important to note that different cell lines are able to demonstrate opposite preferences for substrate stiffness. For example, A549 lung carcinoma-derived epithelial cells show a positive correlation between spreading and substrate stiffness: the stiffer the substrate, the larger the area of cell spreading [31]. Interestingly, the spheroids formed in the presence of Yoda1 had almost similar spreading rates on plastic and glass surfaces. Thus, the presence of Yoda1 during spheroid formation levelled out the substrate-dependent S2/S1 ratio; i.e., in the presence of the chemical agonist of Piezo1 channels, eMSC spheroids became “rigidity independent” [30].

## 3. Materials and Methods

### 3.1. Cells and Reagents

Human endometrial mesenchymal stem cells (eMSCs) isolated from desquamated endometrium of menstrual blood (eMSC line No. 2804) were previously characterized in the Department of Intracellular Signaling, Institute of Cytology, Russian Academy of Sciences (St. Petersburg, Russia) [18]. Particularly, eMSCs met minimal criteria of the International Society for Cell Therapy for multipotent MSCs [32]. Particularly, eMSCs expressed mesenchymal surface markers (CD13, CD29, CDD44, CD73, CD90, CD105), and were negative for hematopoietic markers (CD11b, CD34, CD45, CD117, CD130, HLA-DR class 2) and capable of differentiation in the adipogenic and osteogenic directions [18]. eMSCs were cultured in DMEM/F12 medium (Gibco, Waltham, MA, USA) with 10% fetal bovine serum (Biowest, Nuaillé, France) and antibiotic gentamicin (80 µg/mL, Biolot, St. Petersburg, Russia) in humidified incubator at 37 °C and 5% CO_2_. Cell passaging was performed twice a week at a ratio of 1:3 using a 0.25% trypsin: EDTA solution (Gibco, Waltham, MA, USA). Cells at passages from 9 to 13 were used in the experiments.

Selective Piezo1 activator Yoda1 (2-(5-{[(2,6-dichlorophenyl)- methyl]sulfanyl}-1,3,4-thiadiazol-2-yl) pyrazine, Tocris, UK, Cat. 5580) was dissolved in dimethylsulfoxide (DMSO, Sigma-Aldrich, St. Louis, MO, USA) to obtain a stock solution of 10 mM. Working solutions of Yoda1 (10 µM) were prepared prior to the experiments. As a control, an equal amount of vehicle (0.1% DMSO) was used.

### 3.2. Spheroid Formation and Viability Assay

The eMSC spheroids were formed from a suspension of conventional eMSC culture using the hanging drop method. Firstly, eMSCs were trypsinized, collected in culture medium and counted using a Countess II Automated Cell Counter (Thermo Fisher Scientific, Waltham, MA, USA). The cell concentration in suspension was adjusted to 2 × 10^5^ cells/mL (equal to 7.000 of cells in 35 µL volume of hanging drop). Drops were placed on the cover of 10 cm Petri dishes, overlaid and transferred to a CO_2_ incubator. Cells under the action of surface tension and gravity aggregated in hanging drops for 48–72 h. eMSCs in spheroids retained all main properties of eMSCs in 2D culture including differentiation potential, and expression of CD markers, except for CD146 [33]. The process of spheroid formation was monitored and captured every 24 h using an upright digital microscope with a CCD camera.

To investigate the effect of Piezo1 activation on spheroid formation, we used 10 µM Yoda1, a selective chemical activator for Piezo1 channels. Yoda1 was added to cell suspension before the formation of hanging drops, and the cells were exposed to Yoda1 during the spheroid formation process (48–72 h). After 48–72 h, the images of the spheroids formed in control and in the presence of Yoda1 were captured using a CCD digital camera. The spheroid area and shape descriptors (circularity and aspect ratio) were quantified using ImageJ v1.47. The interpretation of the two latter parameters is as follows: the closer its circularity to 1, the closer the shape of the spheroid is to the ideal circle. The higher the aspect ratio, the more elongated and irregular is the shape of the spheroid.

**Figure 8:**
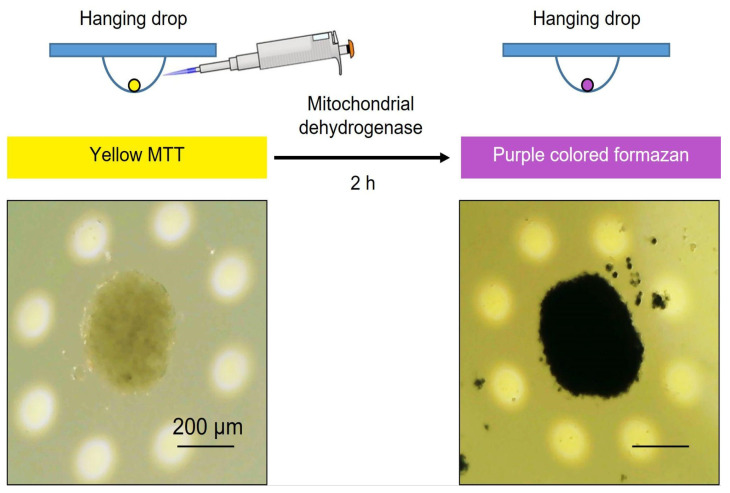
Spheroid viability assay in the hanging drops using MTT reagent. Shown is the representative spheroid before (at the left) and 2 h after (at the right) MTT addition to the drop. Note the dark color of the spheroid that indicates the processing of yellow MTT to purple formazan product by mitochondrial dehydrogenases of viable cells.

To probe the viability of the cells during spheroid formation in control and in the presence of Yoda1, we utilized MTT dye (Cat. No GT4101, ServiceBio, Wuhan, China) based on the ability of living metabolically active cells to process MTT (3-(4,5-dimethylthiazol-2-yl)-2,5-diphenyltetrazolium bromide) into the water-insoluble violet crystals of colored product formazan. Every 24 h, 10 μL of MTT was added to several (3–5) droplets containing the cells to monitor cell viability at various stages (24, 48, and 72 h) after the start of 3D structure formation. After 2 h from the addition of MTT, we captured the images of the spheroids using an upright microscope and CCD camera (Figure 8): the presence of a dark staining of the cells indicated their viable status (see also Figure 3).

### 3.3. RNA Extraction and cDNA Synthesis

Total RNA extraction (from eMSCs cultured as cell monolayer or spheroids) was performed using an RNA Solo kit (Cat. No BC034T, Evrogen, Moscow, Russia). Briefly, eMSCs were trypsinized with 0.25% trypsin-EDTA solution and counted using a Countess II Automated Cell Counter. For total RNA extraction, 3 × 10^6^ cells and ~420 spheroids were taken (equal to 3 × 10^6^ cells as one spheroid contains approximately 7000 cells). All steps of RNA extraction, including DNAse treatment, were performed according to the manufacturer’s instructions. RNA concentration was quantified using GeneQuant 1300 Spectrophotometer (GE Healthcare, Chicago, IL, USA). Complementary DNA (cDNA) was synthesized from 1 µg of total RNA in a 20 µL final volume using an MMLV RT kit (Cat No. SK022, Evrogen, Moscow, Russia) according to the manufacturer’s instructions.

### 3.4. Reverse Transcription (RT) and Quantitative Polymerase Chain Reaction (qPCR)

Before qPCR, all primers were tested on cDNAs to determine the optimal conditions and expected product sizes using conventional RT-PCR. The cycling conditions were as in previous studies [34]. The number of cycling conditions was 35 cycles. qPCR was performed in the presence of intercalating dye SYBR Green I and the reference dye ROX (Sintol, Moscow, Russia). The qPCR reactions were conducted in a total volume of 25 µL with 20 ng of the cDNA sample with six technical replicates on a CFX96 Touch Real-Time PCR detection system (Bio-Rad, Irvine, CA, USA). All experiments were performed in triplicate. *HPRT1* (hypoxanthine phosphoribosyltransferase 1) and *TBP* (TATA-box binding protein) housekeeping genes were selected as stable reference genes for mesenchymal stem cells [35]. The primer sequences of target (*PIEZO1*) and reference genes are listed in Table 1. The amplification steps included cDNA denaturation at 95 °C for 5 min, and 45 cycles of 95 °C for 30 s, 57 °C for 30 s, and 72 °C for 30 s, followed by fluorescence plate reading. Target (*Piezo1*) gene expression was normalized to the expression of housekeeping genes *HPRT1* and *TBP*, and calculated using the 2^−ΔΔCT^ method.

### 3.5. Immunofluorescence

Specific antibodies against the extracellular region of Piezo1 (Proteintech, Rosemont, IL, USA, Cat. No 15939-1-AP, RRID: AB_2231460) were used to visualize the channels in the plasma membrane of eMSC spheroids. The spheroids were seeded on glass coverslips (2 h before the experiments) coated with poly-DL-lysine (Sigma-Aldrich, St. Louis, MO, USA) and fixed with 3.7% paraformaldehyde for 10 min. Non-specific staining was blocked by incubating the cells in a 1 × phosphate buffered saline (PBS) buffer containing 10% goat serum for 1 h at room temperature. The spheroids were then incubated with primary anti-Piezo1 antibodies (at 1:100 dilution) overnight at 4 °C followed by incubation with secondary fluorescent antibodies (1:200 dilution, goat-anti-rabbit-Cy3, Thermo Fisher, Waltham, MA, USA) in the dark for 1 h. The cell nuclei were counterstained with 0.05 µg/mL 4′,6-diamidino-2-phenylindole (DAPI, Sigma-Aldrich, St. Louis, MO, USA) at room temperature for 30 min. After each step of staining, the cells were washed 3–5 times with 1× PBS. After the staining, the glass coverslips were mounted on glass slides using the Vectashield mounting medium (Vector Laboratories, Newark, CA, USA) to minimize photobleaching. The images of the spheroids were acquired on an Olympus FV3000 confocal microscope using 40×/1.3 NA oil immersion objective. The fluorescence was excited at 405 and 561 nm for DAPI and Cy3, respectively. The resulting images were merged and processed with FIJI 1.54f software (NIH, Bethesda, CA, USA). As a control, spheroids incubated in the absence of primary antibodies were used; no staining was observed under these conditions (Supplementary Figure S2).

### 3.6. Ca^2+^ Measurements

Ca^2+^ imaging setup based on an inverted fluorescent microscope (Micromed I LUM, Micromed, St. Petersburg, Russia) with a 20×/0.4 NA Plan Apochromat objective, high-sensitive CCD camera (1360 × 1024 pixels, UHCCD01400KPB, Touptek Photonics, Hangzhou, China) and ToupView 4.11 controlling software (Touptek Photonics, China) was used to register the fluorescence of Fluo8-AM Ca^2+^ probe (Cat. No 21080, AAT Bioquest Inc., Pleasanton, CA, USA) similar to the method previously described [25]. Briefly, eMSC spheroids were plated onto poly-DL-lysine-coated glass coverslips (for 2 h) and loaded with 5 µM cell-permeable Fluo8-AM dye for 45 min (at 37 °C and 5% CO_2_) in 2Ca solution containing (in mM) 150 NaCl, 6 KCl, 1 MgCl_2_, 2 CaCl_2_, and 10 HEPES/TrisOH, pH of 7.3. After 45 min, the dye was washed out, and the spheroids were incubated for a further 15 min to ensure complete de-esterification of the acetoxymethyl (AM) groups. Following this, the spheroids were transferred to an experimental chamber containing a 2Ca solution. The background level of fluorescence was recorded for 30–40 s, and then 10 μM Yoda1 in 2Ca was added to stimulate calcium entry through Piezo1. Ionomycin (5 µM), a Ca^2+^ ionophore, was applied at the end of each experiment to elicit maximal Ca^2+^ influx into the cells. Videos of Ca^2+^ responses of the spheroids were recorded at 3 frames per second, and then converted to image stacks using FIJI software for quantification. Several cells on the edge of the spheroid were selected using the Freehand selection tool, and each cell was used as an individual region of interest (ROI) for fluorescence measurements. A minimum of 7–9 ROIs were analyzed in each spheroid. Calcium imaging experiments with Yoda1 were repeated on at least 3 spheroids independently. To compare the fluorescence intensities between the cells, the basal level of fluorescence (before Yoda1 application) and maximal fluorescence intensity after Yoda1 were normalized to the fluorescence intensity after application of ionomycin (maximal Ca^2+^ entry) to eliminate the possible differences in the efficiency of cell loading with Fluo8 Ca^2+^ probe.

### 3.7. Spheroid Spreading Assay

**Figure 9:**
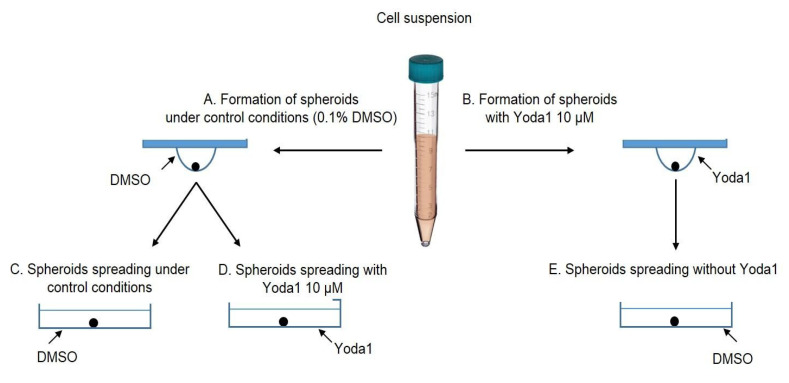
Experimental protocols used in the spheroid assays. Spheroids were formed from cell suspension under control conditions (0.1% DMSO) (**A**) and (**B**) in the presence of 10 µM Yoda1. Then, the control spheroids were plated into wells in 0.1% DMSO (**C**) or in 10 µM Yoda1 (**D**). Spheroids formed in the presence of Yoda1 were plated in 0.1% DMSO (**E**) (Yoda1 was washed out).

Spheroid spreading is the process of cell spreading from the spheroids plated on the flat surface. To determine the role of Piezo1 activity in eMSC spheroid spreading, each droplet with the formed spheroid was manually transferred to a separate well of a 96-well plate, and the spheroids were allowed to attach to the surface for 2 h. Since one well of the plate contained only one spheroid, we analyzed each spheroid individually. Then, the reagents (10 µM Yoda1 or 0.1% DMSO) were added to the wells of the plate (see Figure 9C,D). Spheroids that were exposed to Yoda1 during their formation were spread under Yoda1-free conditions: the cell medium was completely replaced to full culture media with 0.1% DMSO as a vehicle (Figure 9E). The images of the spheroids were captured using an inverted microscope and CCD camera after spheroid attachment (2 h) and 24 h from the beginning of the experiment. From these images, the areas of the spheroids together with the cells that migrated from the spheroid mass were determined using the Measure command of FIJI software. Then, the rate of the spheroid spreading was determined as S2/S1 ratio, where S1 was the area of the initial spheroid (at 2 h), and S2 was the area of the spheroid and the migrated cells after 24 h.

To compare the spheroid spreading rates on the surfaces of different stiffness and to determine the effect of selective Piezo1 activation on this process, the spheroids formed in control (Figure 9A,C) and in the presence of Yoda1 (Figure 9B,E) were seeded on round borosilicate glass coverslips (0.13–0.16 mm thickness, 18 mm diameter, Cat. No. CB00180RA1, Menzel-Glaser, Braunschweig, Germany) that were placed in the wells of 24-well plates. The rate of spheroid spreading was calculated as described above.

### 3.8. Statistics

The statistical analysis was performed in GraphPad Prism 8.0 (GraphPad Software, Boston, MA, USA), *p* < 0.05 was considered significant. Before the analysis, the data were tested for Gaussian distribution (Shapiro–Wilk test), equality of standard deviations (S.D.) and the presence of outliers. The detected outliers (if any) were excluded from the datasets. For comparison of two experimental groups, two-sample unpaired Student’s *t*-test or Mann–Whitney test were used (for Gaussian or non-Gaussian distributions, respectively). In the case of a non-equal S.D., a *t*-test with Welch’s correction was used. The number of experimental repeats, specific statistical tests used for data comparison, and *p*-values are indicated in the corresponding figure legends.

## 4. Conclusions

In the present study, we investigated the effect of selective Piezo1 activation on the formation and spreading of eMSC spheroids. The presence of endogenous Piezo1 ion channels in the spheroids was confirmed using molecular biology techniques supplemented with Ca^2+^ imaging functional assay. Despite the decrease in *PIEZO1* mRNA, the eMSCs in spheroids retained a significant level of Piezo1 in the plasma membrane that could be activated by a selective Piezo1 agonist, Yoda1. Yoda1 affected both the processes of spheroid formation and spreading: the spheroids formed in the presence of Yoda1 were smaller and of irregular shape, and possessed lower rates of spreading, compared to control. The addition of Yoda1 to the culture media also resulted in the decrease of the spreading of spheroids formed under Yoda1-free conditions (control spheroids). Importantly, Yoda1 eliminated the differences in spheroid spreading rates on plastic and glass surfaces, thus confirming the role of Piezo1 as a sensor of the mechanical properties of the substrate. The results demonstrate several Piezo1-dependent reactions that could be modulated by selective Piezo1 activation in eMSC spheroids.

## Figures and Tables

**Table 1:** Primer sequences (5′ to 3′) used for qPCR analysis. HPRT1 and TBP encoding hypoxanthine phosphoribosyltransferase 1 and TATA-box binding protein, respectively, were used as reference genes.

Gene	Primer Sequence
*Piezo1*	forward: ACTTTCCCATCAGCACTCGG; reverse: CCAAGCAGTCCTTGAGACCC
*HPRT1*	forward: TGACACTGGCAAAACAATGCA; reverse: GGTCCTTTTCACCAGCAAGCT
*TBP*	forward: CACGAACCACGGCACTGATT; reverse: TTTTCTTGCTGCCAGTCTGGAC

## Data Availability

The raw data supporting the conclusions of this article will be made available by the authors on request.

## Supplementary Materials

The following supporting information can be downloaded at: https://www.mdpi.com/article/10.3390/ijms26062474/s1.
