# Supplementary material for: Piezo1 Ion Channels Regulate the Formation and Spreading of Human Endometrial Mesenchymal Stem Cell Spheroids
Source: Int J Mol Sci. 2025 Mar 10;26(6):2474. doi: 10.3390/ijms26062474 (PMC11942067; doi:10.3390/ijms26062474)
Supplement: Supplementary file 1 [file ijms-26-02474-s001.zip › Supplementary Figure 3 Legends.pdf]

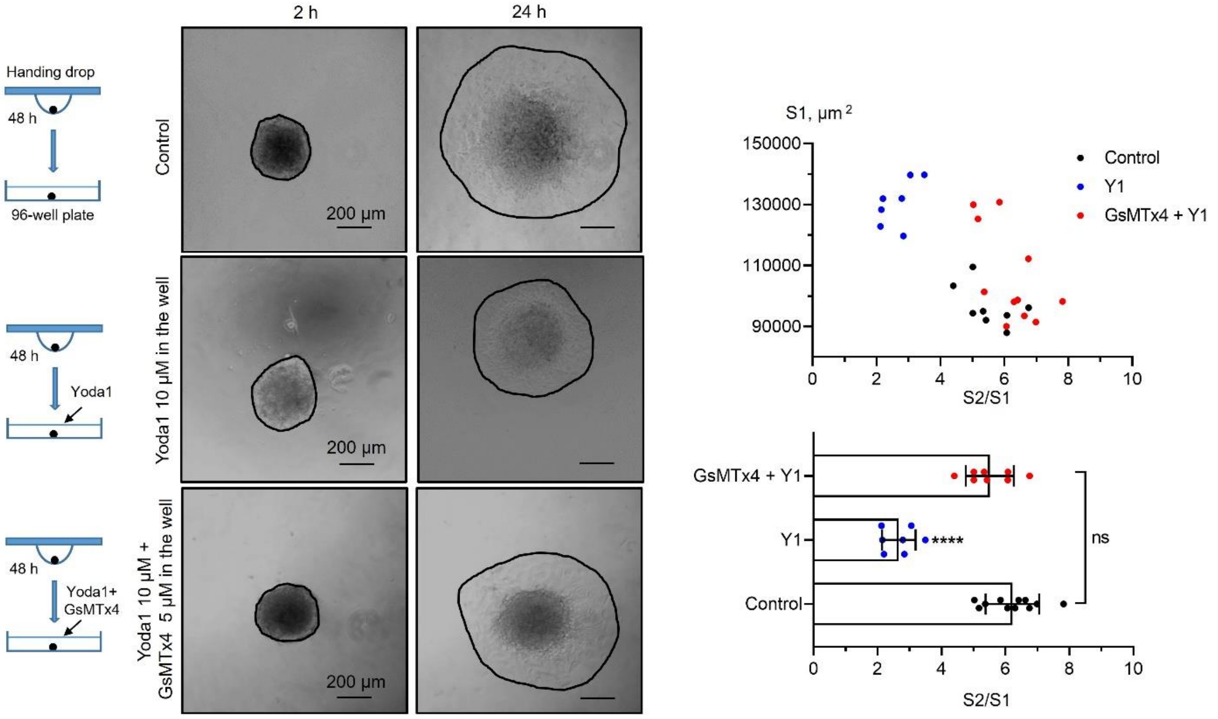

Figure S3. GsMTx4 abolished the effect of Yoda1 on eMSC spheroid spreading. GsMTx4 was purchased from Tocris (Cat No. 4912, Tocris Bioscience, Bristol, United Kingdom). The spheroids were pre-incubated with 5 µM of GsMTx4 for 10 minutes to allow toxin binding, and then 10 µM Yoda1 was added to the same wells (without GsMTx4 wash-out). The spheroid reactivation rates were calculated similarly to other experiments (see Materials and Methods and Results and Discussion). ns - non-significantly different, ****$p<0.0001$, compared to control and to GsMTx4+Yoda1, unpaired t-test.
