# Supplementary figures and images for: Piezo1 Ion Channels Regulate the Formation and Spreading of Human Endometrial Mesenchymal Stem Cell Spheroids
Source: Int J Mol Sci. 2025 Mar 10;26(6):2474. doi: 10.3390/ijms26062474 (PMC11942067; doi:10.3390/ijms26062474)

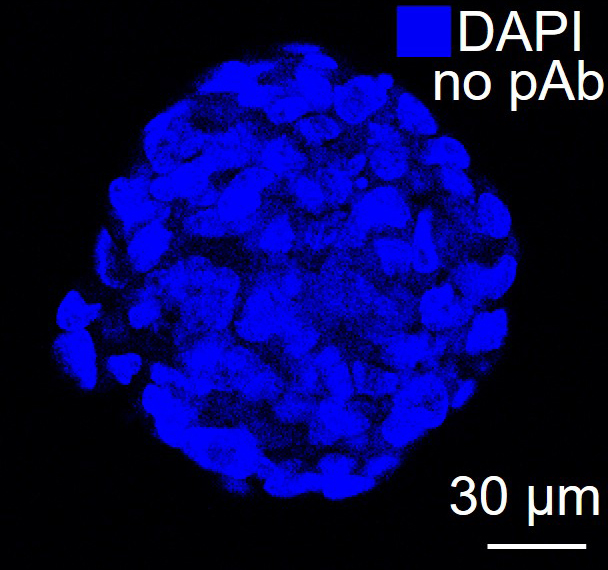

Supplement: Supplementary file 1 [file ijms-26-02474-s001.zip › Figure S1.jpg]

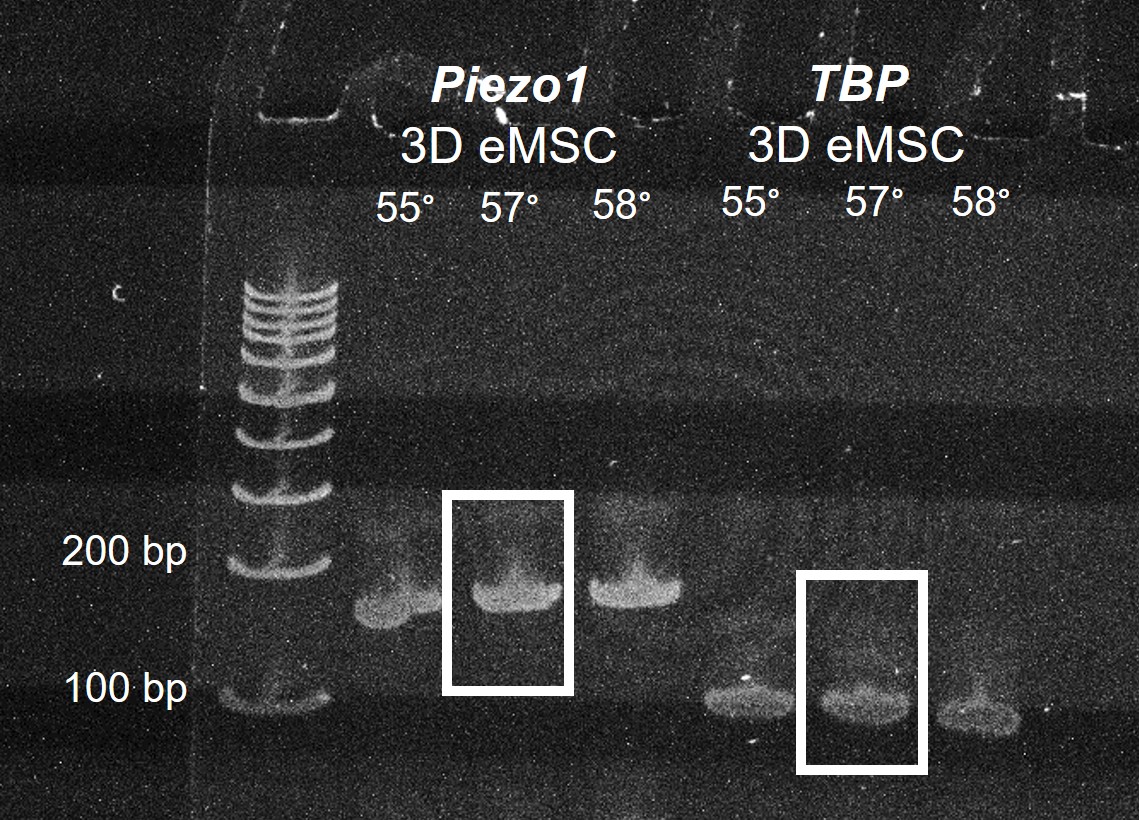

Supplement: Supplementary file 1 [file ijms-26-02474-s001.zip › Figure S2_Gel1.jpg]

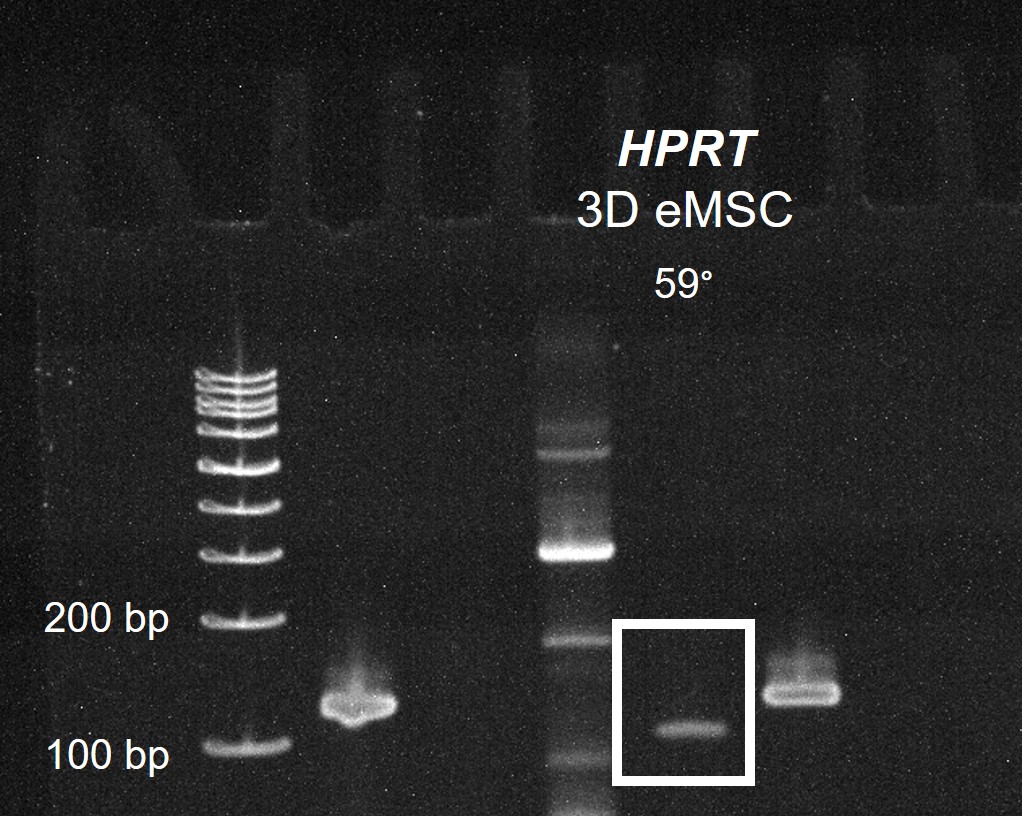

Supplement: Supplementary file 1 [file ijms-26-02474-s001.zip › Figure S2_Gel2.jpg]

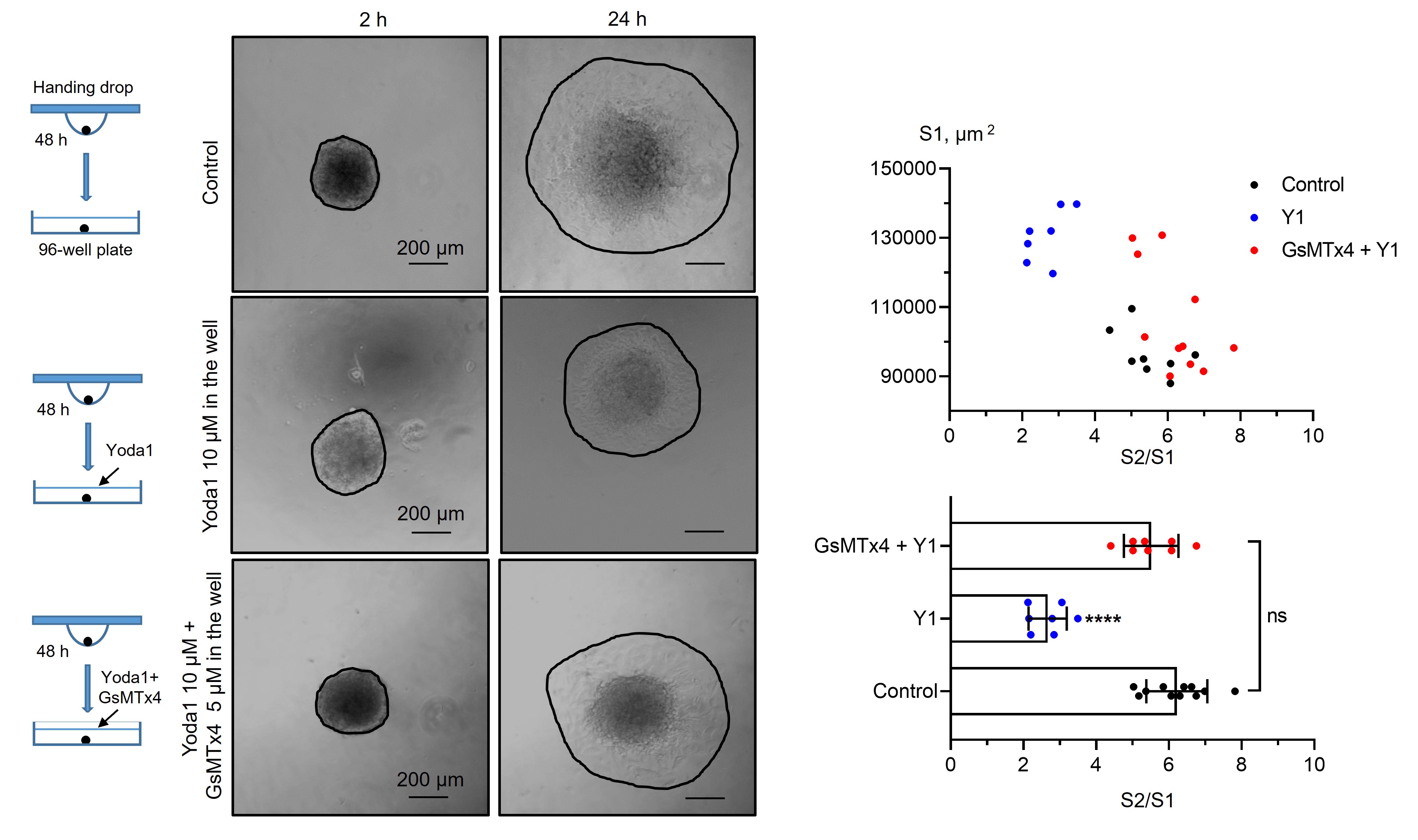

Supplement: Supplementary file 1 [file ijms-26-02474-s001.zip › Figure S3.jpg]
